# From Pandemicity to Endemicity: The Journey of SARS-CoV-2

**DOI:** 10.1007/s44197-022-00046-4

**Published:** 2022-06-15

**Authors:** Jaffar A. Al-Tawfiq, Dinh-Toi Chu, Van-Thuan Hoang, Ziad A. Memish

**Affiliations:** 1 Infectious Disease Unit, Specialty Internal Medicine, Johns Hopkins Aramco Healthcare, Dhahran, Saudi Arabia; 2 Department of Medicine, Indiana University School of Medicine, Indianapolis, IN USA; 3 Department of Medicine, Johns Hopkins University School of Medicine, Baltimore, MD USA; 4 Center for Biomedicine and Community Health, International School, Vietnam National University, Hanoi, Vietnam; 5 Thai Binh University of Medicine and Pharmacy, Thai Binh, Vietnam; 6 Director Research and Innovation Centre, King Saud Medical City, Ministry of Health and College of Medicine, Alfaisal University, Riyadh, Kingdom of Saudi Arabia; 7 Hubert Department of Global Health, Rollins School of Public Health, Emory University, Atlanta, GA USA

Since the emergence of the Severe Acute Respiratory Syndrome Coronavirus 2 (SARS-CoV-2), scientists around the globe had raced to produce multiple effective vaccines using old and brand-new platforms. International regulatory agencies developed synchronized and expedited review and approval processes. These vaccines, in addition to natural immunity from infection had contributed to providing the needed immunity to prevent severe disease and mortality. However, the current vaccines do not seem to prevent asymptomatic or mild infection [1]. The contribution of asymptomatic infections to the pandemic was described at an early stage of the disease [2]. In the 2 ½ years’ time-span under the impact of different waves of this global pandemic, SARS-CoV-2 to date had infected an estimated 430,257,564 confirmed cases, including 5,922,047 deaths as reported to the World Health Organization (WHO) and a total of 10,407,359,583 vaccine doses had been given globally [3]. The ultimate hope is that SARS-CoV-2 with its continuous mutation will become less impactful and transform into an endemic state and the world would treat it as the common cold or annual seasonal influenza.

Certain countries like Singapore with a vaccination rate reaching 80%, had resolved into an endemic state [4, 5]. This was done cautiously with the opening of the economy and lifting of social restrictions. This cautious opening however had resulted in another problem of having increased Coronavirus Disease 2019 (COVID-19) cases in long-term care [6]. This increase may indicate the waning immunity as well as less adherence to non-pharmacologic interventions that resulted in the reduction of many respiratory viral infections like influenza. Such a state of endemicity would be welcomed if the SARS-CoV-2 becomes less virulence over time [7], causes less severe disease and lower death rates. In a survey of immunologists, 90% of the respondents expected SARS-CoV-2 to become endemic and about 33% said that it is possible to eliminate SARS-CoV-2 from a few regions [8]. It was suggested that re-infection with SARS-CoV-2 in an endemic situation is probable to occur 3 months to 5·1 years after the peak antibody response and a median of 16 months, which is less than half the duration for the other human endemic coronaviruses [9]. Common human respiratory coronaviruses, NL63 or 229E, have a seroprevalence of 65–75% among those of 2.5–3.5 years of age [10]. The rate of reinfection with OC43 may occur due to genetic substitution in the spike protein [11]. Persistence of antibodies among the 2002 SARS-CoV was described for a mean of 2 years in one study [12], sustained for > 150 days in another study [13], and detected > 200–240 days [14, 15].

The emergence of the SARS-CoV-1 in 2003 was followed by the complete disappearance of the virus within approximately 3–4 months, however, the pandemic H1N1 emergence in 2009 was associated with persistent infection among the human population. The scenario is not yet clear with SARS-COV-2, but a few possibilities remain (Fig. 1). However, it seems that an equilibrium is being reached between the SARS-CoV-2 and the human population through vaccination and natural infection. However, persistence of the pockets of susceptible individuals could lead to the further emergence of variants [1]. The emergence of different variants of concern such as delta and the Omicron is of particular importance [16]. Omicron had caused global additional waves and had been associated with less severe disease especially among vaccinated individuals [17, 18]. It is feared that continued mutation may occur due to the sustained transmission between humans as well as between humans and animals [19]. The importance of vaccines is the ability to cause less severe disease and less transmission through less susceptible individuals with transition from pandemic to endemic with a stable number of infections in the population [20]. The continuing low-level occurrence of SARS-CoV-2 during endemicity would ensure the maintenance of the needed immunity among the populations [21]. Thus, it is expected that the SARS-CoV-2 would become part of the new-normal of our lives similar to other infectious diseases such as other human coronaviruses, tuberculosis and influenza. However, the endemic disease does not equate an end of the virus [22] but the ability to cause a steady-state of infection but may still cause significant morbidity among immunocompromised hosts. Therefore, as the world returns to the new normal and lives with the COVID-19, people at risk of severe illness need to be monitored, cared for, and prevented to reduce mortality. International Public Health Agency (PHA) like Coalition for Epidemic Preparedness Innovations (CEPI) and United Sates National Institute of Health (NIH) had committed a huge fund exceeding $250 million for the development of a new universal Coronavirus vaccine that if it succeeds would put SARS-CoV-2 pandemic out and minimize its impact on the susceptible and immunocompromised in the future. The development of the intra-nasal COVID-19 vaccine may also lead to sterilizing immunity and thus prevent further transmission of the virus [23, 24].

**Fig. 1:**
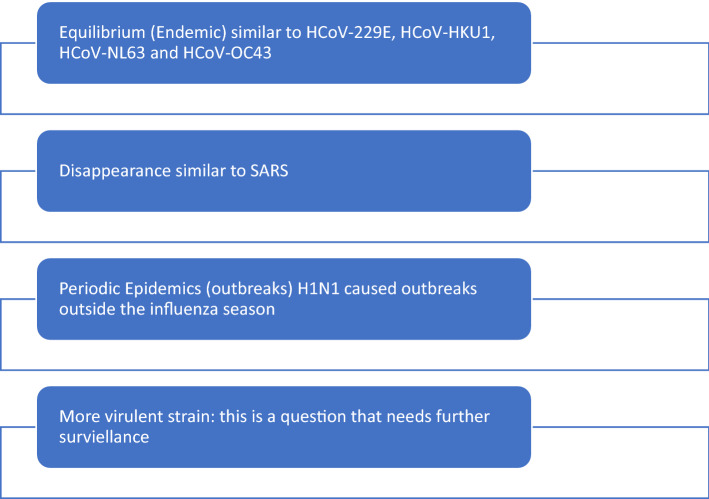
Possible Projected SARS-CoV-2 EventsF

In conclusion, the future of the COVID-19 pandemic may follow the development of endemicity of SARS-CoV-2 and may be associated with epidemics in communities wit low vaccination. It is unlikely that SARS-CoV-2 will disappear completely.

## Data Availability

Not applicable.

## Abbreviations

SARS-CoV-2: Severe Acute Respiratory Syndrome Coronavirus 2
WHO: World Health Organization
COVID-19: Coronavirus Disease 2019
PHA: Public Health Agency
PHA: International Public Health Agency
CEPI: Coalition for Epidemic Preparedness Innovations
NIH: National Institute of Health
